# Scaling logical density of DNA storage with enzymatically-ligated composite motifs

**DOI:** 10.1038/s41598-023-43172-0

**Published:** 2023-09-25

**Authors:** Yiqing Yan, Nimesh Pinnamaneni, Sachin Chalapati, Conor Crosbie, Raja Appuswamy

**Affiliations:** 1 Data Science Department, EURECOM, Biot, France; 2 Helixworks Technologies, Cork, Ireland

**Keywords:** Biotechnology, Synthetic biology, Computer science

## Abstract

DNA is a promising candidate for long-term data storage due to its high density and endurance. The key challenge in DNA storage today is the cost of synthesis. In this work, we propose *composite motifs*, a framework that uses a mixture of prefabricated motifs as building blocks to reduce synthesis cost by scaling logical density. To write data, we introduce Bridge Oligonucleotide Assembly, an enzymatic ligation technique for synthesizing oligos based on composite motifs. To sequence data, we introduce Direct Oligonucleotide Sequencing, a nanopore-based technique to sequence short oligos, eliminating common preparatory steps like DNA assembly, amplification and end-prep. To decode data, we introduce Motif-Search, a novel consensus caller that provides accurate reconstruction despite synthesis and sequencing errors. Using the proposed methods, we present an end-to-end experiment where we store the text “HelloWorld” at a logical density of 84 bits/cycle (14–42× improvement over state-of-the-art).

## Introduction

The growing adoption of Big Data Analytics and Artificial Intelligence has led to an explosion in the rate of data generation. A recent survey by the International Data Corporation reports that the digital datasphere is forecast to grow to 125 zettabytes by 2025^1^ and is anticipated to exceed silicon supply in 2040^2^. As traditional storage media is unable to keep pace with the rate of data growth^3^, synthetic DNA has become an attractive archival storage medium due to its high density, longevity, and absence of technical obsolescence compared with electronic media^4–8^.

In most prior work on DNA-based digital storage^5,7,9,10^, DNA synthesis is based on phosphoramidite chemistry^11^, a technology that has been optimized over several decades to perform highly-accurate, base-by-base synthesis of short DNA strands by making phosphodiester bonds between nucleotides. There are three Key Performance Indicators (KPIs) that can be used to evaluate the efficiency of DNA synthesis: (i) bits written per cycle (also called *logical density*)^12,13^, (ii) bits written per oligo, and (iii) coupling reactions per oligo. The efficiency of writing data to DNA depends on the number of synthesis cycles (*x*) to grow the strand and available repeating units (*m*) for addition at each cycle. The information capacity of the oligo (*N* bits) can be derived as

1 $$\begin{aligned} N (bits) = x \times log_2m \end{aligned}$$

While base-by-base synthesis methods can perform 200 or more coupling cycles(*x*), the number of available subunits to add at each cycle is four (nucleotides), thereby limiting bits per synthesis cycle to two, and the information capacity of an oligo to a few hundred bits. While the quality, quantity, cost, and rate of DNA synthesis provided by base-by-base chemistry is suitable for biological research, it is far from ideal for the DNA storage use case. This has resulted in synthesis emerging as a major bottleneck in DNA storage.

In this work, we introduce the *composite motifs* framework to scale logical density well beyond the limit of 2 bits per synthesis cycle. Our inspiration stems from recent work on DNA storage involving the introduction of composite letters, also known as degenerate bases^12,13^. These approaches leverage each position of a sequence containing a combination of all four DNA nucleotides in predefined ratios to increase logical density to 6.38 bits per cycle. However, these methods increase read cost, as they require a substantially higher sequencing coverage of 100–250$\times$ to ensure complete data recovery, due to the utilization of composite ratios.

Our composite motif approach extends the work on degenerate bases by using short oligonucleotide sequences, also referred to as motifs^14,15^, as building blocks. These motifs are drawn from a fixed library for assembling longer oligos. The use of a fixed library of motifs similar to a typesetting press can also simplify miniaturization and automation. The composite motif framework builds on the benefits of motif-based DNA storage^14,15^, and further improves logical density by exploiting sequencing multiplicity inherent in DNA synthesis by encoding data using a combination of motifs rather than individual motifs.

In this work, we present a DNA storage system that leverages composite motifs to achieve an order of magnitude enhancement in logical density compared to existing state-of-the-art solutions. Our end-to-end prototype system introduces enzymatic motif ligation techniques for composite encodings, enhancing the efficiency and throughput of the DNA synthesis process within the write pipeline. Furthermore, we employ a Nanopore-based approach for motif readout, which allows for the direct sequencing of short oligos without the need for common preparatory steps like DNA assembly, amplification, and end-prep. This, coupled with our alignment-based motif decoding techniques, streamlines the DNA read pipeline, offering a scalable and efficient solution for DNA sequencing.

## Results

### Composite motifs as building blocks for DNA storage

A composite motif is a representation of a position in an oligo sequence that uses a combination of motifs drawn from a fixed motif library to encode data. For example, assuming a library of 32 motifs, and a combination factor of four, there are $C(32, 4) = 35960$ possible unique combinations with which we can encode 15 ($log_2{35960}$) bits of data per composite motif. Composite motifs increase logical density by expanding the motif library using combinations of motifs without increasing the volume of motifs. As current synthesis platforms already use a high degree of sequence multiplicity (multiple copies of DNA molecules are synthesized per oligo), composite motifs can also be integrated into current platforms without any extra cost as they can exploit sequence multiplicity to scale logical density. Higher logical density also leads to a reduction in the length of DNA required to store the same amount of data, alleviating issues related to long oligo synthesis.

**Figure 1:**
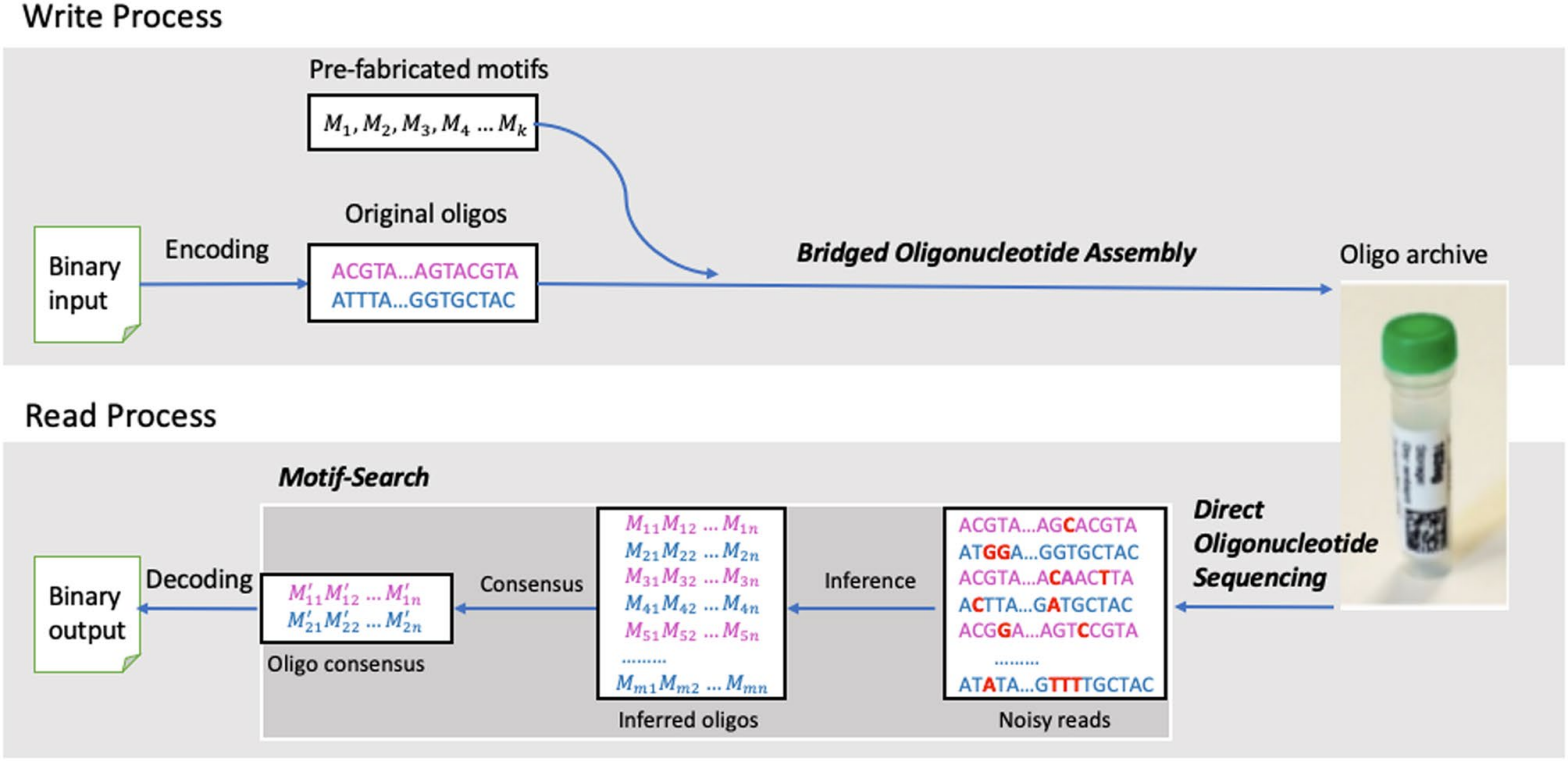
Composite-motif-based data write and read pipeline.

In order to demonstrate the feasibility of using composite motifs, we developed a DNA storage system that uses composite motifs as building blocks. Figure 1 presents the read/write pipeline of our system. On the writing side, digital data is encoded into oligos containing composite motifs using a motif encoder. Writing a composite motif at any given position of an oligo sequence is done by mixing multiple motifs during the synthesis procedure to synthesize multiple DNA molecules that contain the corresponding combinations of motifs using *Bridged Oligonucleotide Assembly* (BOA) (“Bridged Assembly of Composite Motifs” Section). On the reading side (Fig. 1), we read composite motifs by amplification-free sequencing of multiple DNA molecules using *Direct Oligonucleotide Sequencing* (DOS) (”Direct Nanopore Sequencing and Error Characterization” Section), and then decode the data using our new motif-based consensus caller called *Motif-Search* (“Inference and Consensus with Motif Search” Section).

### Bridged assembly of composite motifs

#### Encoding

Data was encoded using a library of 96 payload motif sequences and 8 address motif sequences. The sequence design rules for base motifs that are used to derive composite motifs are similar to those of DNA barcode design. Thus, we started with DNA sequences designed in prior work^16^ to select to select 8 motifs for addressing and 96 motifs for composite payloads. Using a combination factor of 32, we developed a composite motif set of $3 \times 10^{25}$ composite motifs (*C*(96, 32)). Thus, each composite motif, and hence, each synthesis cycle, can store 84-bits of data ($log_2{C(96, 32)}$). In order to demonstrate the feasibility of composite motifs, we stored the text “HelloWorld” using our composite-motif-based DNA storage system. As our input text is 10 bytes, a single synthesis reaction with composite motifs was sufficient to store the data. In order to test precision and recall of methods in the read pipeline, we stored the same data on all eight address motifs.

**Figure 2:**
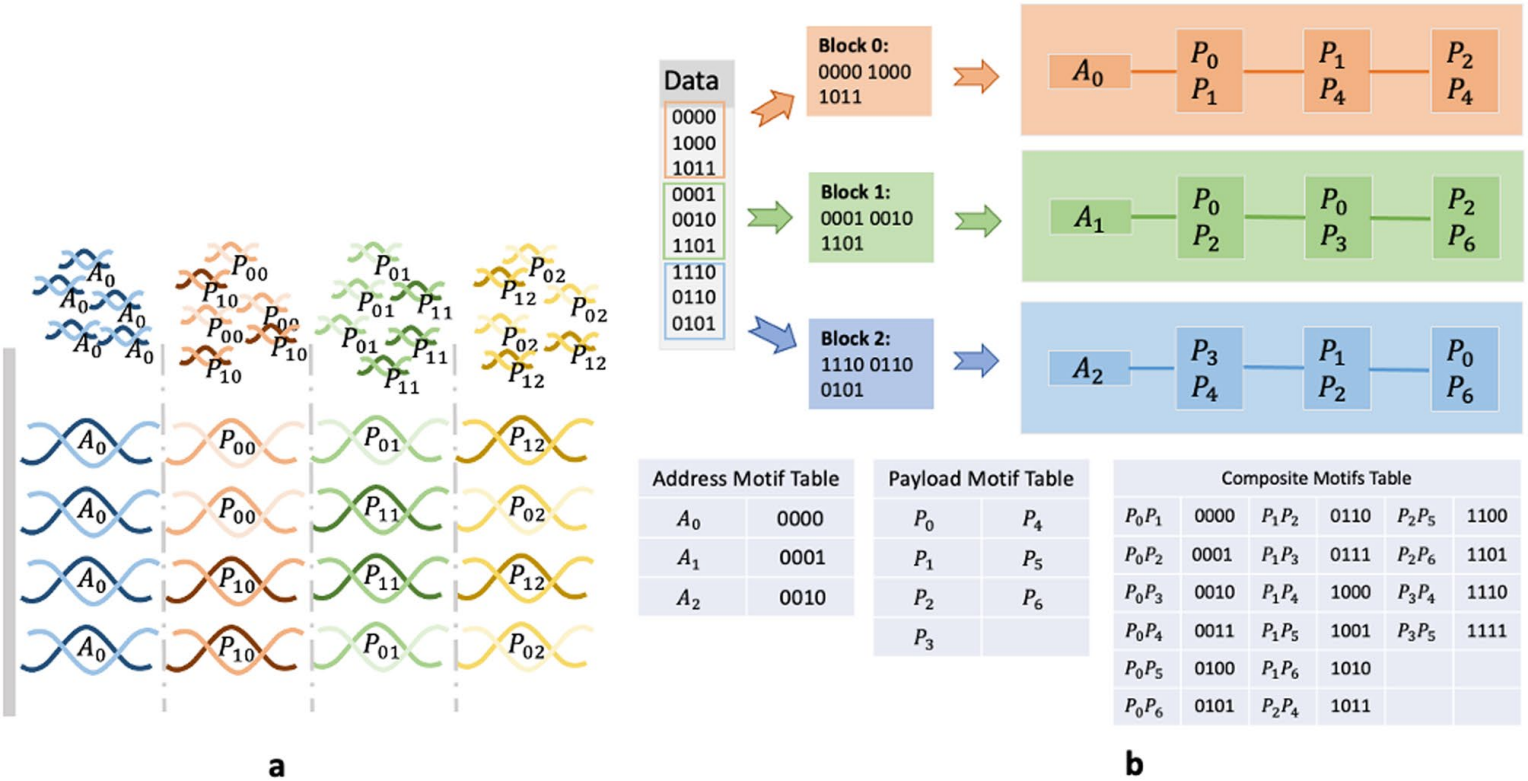
Composite motifs scales the logical density in DNA-based storage. **(a)** In each synthesis cycle, a combination of different payload motifs is introduced into the pool, rather than a uniform type of motifs. For instance, the initial composite motif comprises a blend of $P_{00}$ and $P_{10}$, followed by $P_{01}$ and $P_{11}$ in the second cycle, and $P_{02}$ and $P_{12}$ in the third. Consequently, four different oligos, $P_{00}-P_{01}-P_{12}$, $P_{00}-P_{11}-P_{02}$, $P_{10}-P_{11}-P_{12}$ and $P_{10}-P_{01}-P_{02}$ are synthesized. These four oligos are called a logical sequence, sharing the same address motifs and representing the logical binary data together. **(b)** An example of the encoding process. Given seven payload motifs and a combination factor of two, each composite motifs can represent 21 (*C*(7, 2)) distinct values, effectively encoding four bits. For instance, the initial four bits binary input 0000 is represented as $P_0$ and $P_{1}$. Assuming each oligo has three payload motifs, the 36 bits input data need to be splited into 3 blocks and then each block is transformed to a logical sequence. A: address motif for indexing logical sequences, P: payload motif for encoding actual data.

The size of payload, address motif and combination factor were chosen to provide a balance between complexity and manageability of initial experiment with manual liquid-handling in the lab. With a library size of 96, the maximum combination factor is 48 because *C*(96, 48) is the biggest value among all *C*(96, *i*) where $i \le 96, i \in \mathbb {N_+}$. However, choosing 32 allows us to achieve a high level of complexity (sufficient for our 10-byte “HelloWorld” message) while reducing the challenges associated with manual liquid-handling in the lab. Each oligo synthesis reaction in this method requires 32 dispenses for the composite motif, one dispense for the address motif and bridge oligo, and one dispense for the ligation enzyme buffer. While we designed our encoding to match our experimental needs, it naturally extends to a general purpose encoder(Fig. 2).

#### Bridge oligonucleotide assembly

**Figure 3:**
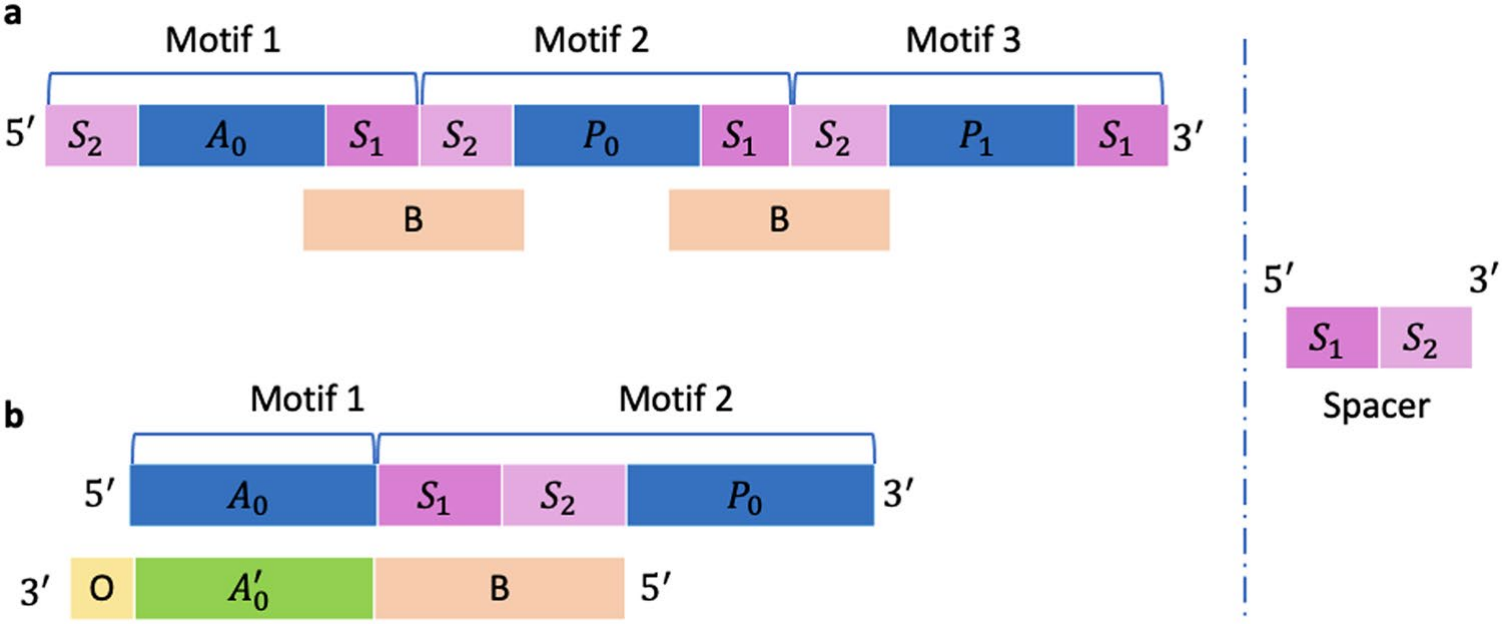
Bridged oligonucleotide assembly. (**a**) The general oligo structure design. (**b**) The experimental oligo structure design. A: address motif, A′: reverse complement of A, P: payload motif, S: spacer, B: bridge, O: overhang.

Each of the eight address motifs along with the composite motif mix of 32 oligos produced a total of 256 oligos. The address motif is repeated in each molecule, while the composite motif is expanded to generate a variant combination using 32 payload motifs. Oligos are synthesized using template-directed ligation. This method utilises single-strand sequences, referred to as *bridge* oligos, to facilitate the ligation of payload motifs to address motifs. In the general case, an oligo would contain one or more address and payload motifs as shown in Fig. 2. As any motif can be ligated with any other, designing bridge oligos for each possibility is suboptimal and not scalable. We solve this problem by using a *spacer* motif. When the motif library is designed, each 25nt motif is extended on both 5′ and 3′ ends with 12nt and 13nt nucleotides from the 3′ and 5′ ends of the spacer motif (Fig. 3a). While this increases the length of each synthesized motif from 25 to 50 nt, it does not affect the number of motifs, and more importantly, it makes it possible to design the bridge oligo to be complementary to a single spacer. By doing so, the bridge oligos can hybridise to the spacer portions at the 3’ and 5’ ends of two payload motifs while the enzyme ligates them. In general, the spacer motif sequence must be carefully chosen to avoid interference with other address/payload motifs, and to ensure proper alignment and enzymatic ligation. The identification of the optimal spacer sequence and any forbidden strings is context-specific to the motif library used and requires both computational modeling and experimental validation. However, the development of a thoughtfully designed motif library is a one-time investment that can be repeatedly reused to encode data in oligos.

For the purpose of our experiment, as we have only 2 motifs per oligo, we modified this by (i) prepending the entire spacer sequence to the 5′ end of each payload motif, and (ii) designing eight (instead of one) bridge oligos, each of which is complementary to both the spacer sequence and one of the eight address sequences (Fig. 3b). By doing so, the eight bridge motifs also double in role as adapters during sequencing. The spacer-extended 32 payload motifs, eight address motifs, and eight bridge oligos were all synthesized base-by-base by Integrated DNA Technologies (IDT). The oligos were synthesized by selecting, annealing and ligating together the corresponding address–payload motif pairs. The inputs to the reaction comprise all motif oligos, bridge oligos, enzymes and ligation buffer. These reactions proceeded to produce ligated oligos through programmed temperature incubation and cycling, where each bridge oligo facilitates the ligation of a specific address motif with a payload motif via complementary annealing. We use the resulting oligo pool to test the feasibility of decoding the identity of motifs from an enzymatically-ligated, Nanopore-basecalled readout.

### Direct nanopore sequencing and error characterization

In the context of DNA data storage, both the cost of DNA sequencing and the time required to read data from DNA molecules are critical factors. Nanopore sequencing, with its single-molecule sensing capabilities, offers a promising avenue for a low-cost, high-speed DNA storage read head. The efficiency of a Nanopore (ONT) flowcell is contingent on the size of the DNA to be sequenced. Sequencing small oligonucleotides leads to a higher number of unoccupied pores over time. ONT’s R9.4 flowcell, for instance, requires a minimum DNA size of 200 bases. Previous DNA storage approaches using Nanopore sequencing have necessitated labor-intensive and time-consuming sample preparation steps for short oligonucleotides. This often involved DNA assembly methods to concatenate five or more DNA storage oligos into a longer fragment, followed by PCR amplification to enhance sequencing throughput and coverage for decoding^17^.

In this work, we demonstrated a method that facilitates the direct sequencing of composite-motif encoded oligonucleotides, bypassing the need for amplification or second-strand synthesis. Our oligonucleotides consist of two motifs, linked by a spacer. We engineered eight bridge oligonucleotides to incorporate the necessary modifications, making the strand ends available for ligation with the ONT sequencing adaptor. Specifically, the (first) bridge oligo is designed to include in its sequence complementary bases to the address oligo, along with an Adenosine (A) overhang. The complementary address motif oligo has a 5′ phosphorylated end. This configuration of the composite-motif encoded oligo, combining the ‘A’ overhang of the bridge with the 5′ phosphorylation of the address region, emulates a double-stranded DNA (dsDNA) end, enabling ready ligation with the AMX sequencing adapters from ONT’s ligation sequencing kit (LSK-109). The AMX adapters were affixed to the oligonucleotides in a 10-min reaction, and sequencing was conducted on an R9.4.1 flow cell for a duration of 4 h. Both Guppy and Bonito basecallers were utilized for basecalling. The sequencing run yielded 27,198 reads, with an N50 of 192 bp. The presence of reads confirms that the oligo modifications introduced during the synthesis step are suitable for ONT sequencing. We assume that only oligonucleotides ligated with the sequencing adaptor would have been able to pass through the pores, and subsequently be basecalled. This approach to direct sequencing of short oligos improved cost and time efficiency. By eliminating the need for traditional preparatory steps and enabling the direct sequencing of composite-motif encoded oligonucleotides, we streamlined the sequencing process of data storage oligos.

**Figure 4:**
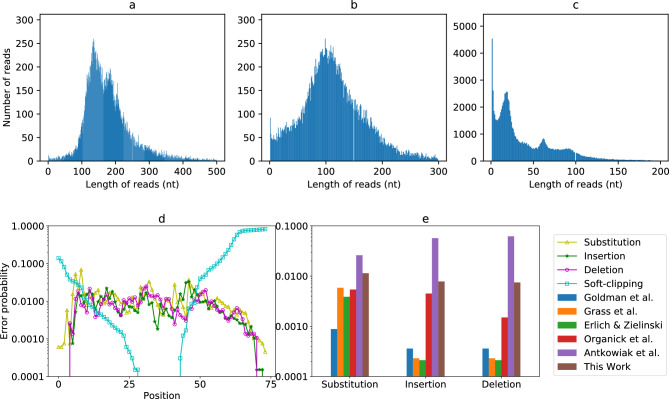
Analysis about the sequenced reads. (**a**) Read length distribution with Guppy basecaller. (**b**) Read length distribution with Bonito basecaller. (**c**) Read length distribution with Bonito basecaller post-processed with SaberSplit . (**d**) The substitution, insertion, deletion and soft-clipping rate per position of Guppy reads. (**e**) Comparison of errors rate with previous work.

Despite having several reads, we found that the reads were low quality. From the read length distribution in Fig. 4a,b, we see that the median read length with Guppy and Bonito is 166 nt and 110 nt. Thus, more than half reads are 48% longer than original oligos as several reads were observed to contain multiple oligos in a single read. On further analysis, we identified wrong event detection by MinKNOW to be the root cause of the problem. When sequencing oligonucleotides on an ONT R9.4 flowcell, the movement of bases through the pore leads to a continual change in current, known as the “squiggle”, that is recorded by MinKNOW. MinKNOW processes the squiggle into reads in real-time, and each read is supposed to correspond to a single strand of DNA. However, as our oligos were below 200 bases, we observed that sequencing our oligos generated low quality reads due to incorrect segmentation by MinKNOW which would earmark empty signals as valid reads, and created reads with merged squiggles for more than one strand of DNA.

Due to the presence of multiple oligos per read, we cannot directly align the reads to the reference oligos. So we did reverse alignment to study error characteristics and coverage distributions. We regard each read as a “reference” and build an index per read. Then, we treat each oligo like a “read”, and align it to each reference. Thus, for each read, we get an alignment file that contains one record per oligo. To identify and retain only good alignments, we filter the alignments using the following criteria: (i) MAPQ > 10 (90% alignment confidence), (ii) all alignments in a read should correspond to one orientation (no mixed forward and reverse alignments), and (iii) there should not be any overlap when multiple oligos are mapped in a single read; only the alignment with the highest alignment score is kept if several alignments overlap each other. With this approach, we get the set of oligos that we can identify assuming we have full knowledge of the original oligos.

Using Minimap2^18^ for reverse alignment (Supplementary Note 2), we computed the substitution, insertion, deletion and soft-clipping rate per position (Fig. 4d). As can be seen, the rate of soft clipping is very high at the extremities (especially 3’ end) due to the very high error rate caused by BOA and DOS. In the middle portion of the read, the rates of error types vary, with no one error type being dominant over others. These results are in sharp contrast to error statistics published in prior work on DNA storage^5,7,10,19,20^, where substitution errors have been shown to be more likely than indel errors, and overall error rates are at least 10$\times$ lower (Fig. 4e and Supplementary Table 1). The only exception is work on photolithographic synthesis^21^, where the error rates reported were also high.

### Correcting event misdetection with SaberSplit

In the real DNA storage scenario, the original reference oligos must be inferred from erroneous reads automatically. Current read clustering and consensus callers used for this purpose assume that a read covers only a single oligo. To be able to use them, we initially analysed the fast5 reads generated from the nanopore sequencing runs by using a tool called SquiggleKit (https://github.com/Psy-Fer/SquiggleKit) to extract the event data. We developed a tool “SaberSplit” (Supplementary Note 1) to split such fast5 reads into multiple shorter reads for basecalling. Using SaberSplit and bonito basecaller, the original reads are chopped to 102,221 shorter reads of median length 25nt as shown in Fig. 4c. Then, we tried to use state-of-the-art clustering programs and position-wise consensus callers^22,23^ to infer the original oligos from both raw Bonito/Guppy reads, and SaberSplit processed reads. However, due to the high error rate, no oligos could be inferred in all cases.

To study SaberSplit processed reads further, we aligned the chopped reads to reference oligos with Minimap2. We compared the alignment statistics for raw Guppy, Bonito and Sabersplit processed reads (Supplementary Table 2). Guppy reads produced the highest number of alignments, with 102% more reads being aligned than Bonito. This could be explained by the fact Bonito is optimized to work better with longer reads, making it less suitable for short ones. Surprisingly, SaberSplit performed the worst with 9.5% fewer reads than even Bonito. This showed us that splitting reads amplifies the error rate and makes the case for a consensus caller that can directly work with raw reads covering multiple oligos.

### Inference and consensus with motif search

To reconstruct the original data from noisy reads, we developed a new reconstruction algorithm called Motif-Search that meets two requirements: (i) guarantee successful recovery despite high error rate, and (ii) directly work with raw, basecalled, Nanopore reads that might contain multiple oligos per read. Motif-Search differs from prior consensus callers that it is structure aware—while other callers view an oligo as a random collection of nucleotides, Motif-Search exploits the fact that our oligos are a collection of payload motifs separated by spacer motifs, with all motifs being drawn from a predefined, finite library. A detailed description of the Motif-Search algorithm is presented in  “Motif-Search Algorithm” Section. Here, we present our analysis results that demonstrate the ability of Motif-Search to accurately infer original oligos.

**Figure 5:**
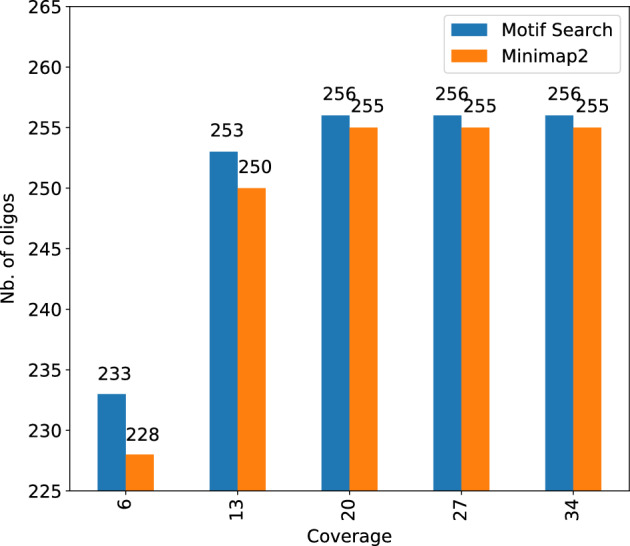
Number of oligos correctly reconstructed. Motif-Search fully recovers all oligos at 20× or higher coverage. Minimap2 misses one oligo even with 34× coverage.

Figure 5 shows the true positive (TP) count (number of inferred oligos that are in the original set) of Motif-Search and Minimap2-based reverse alignment method at various coverage levels (lower sequencing coverage simulated via subsampling reads). It is important to note that Minimap2 needs the original oligos which would not be available in the real DNA storage use case. Thus, Minimap2 results are used as a baseline for comparison rather than a real decoding solution. First, Motif-Search is able to fully recover all oligos at 20× coverage. Reverse alignment misses one oligo even with 34$\times$ coverage. Second, Motif-Search reconstructs more oligos than reverse alignment at all coverage levels. The under-performance of reverse alignment relative to Motif-Search is because all the reads covering the missing oligo had a very poor alignment and were filtered out.

Supplementary Table 3 shows the execution time of Motif-Search and reverse alignment. Both support multi-threaded operation. On a 12-core Intel(R) Core(TM) i9-10920X CPU clocked at 3.50 GHz, 128 GB RAM with a 1TB SATA SSD, Motif-Search is 190–250× faster than Minimap2 due to the fact that Minimap2 needs to build an index for each read and align each oligo to each read while Motif-Search is custom-designed for the motif-based oligo reconstruction use case.

In order to investigate false positive (FP) behavior of Motif-Search and reverse alignment, we increase the motif library size. For a given set of address and payload motifs, we create oligos containing all possible combinations of motifs. For instance, if the motif set size is $64 (address) \times 256 (payload)$, we generate 16,384 possible oligos. We then use Minimap2 to align each oligo to each read. We use the same reads as before which were sequenced from 256 original oligos. As the motif set is expanded, Motif-Search can now report an inferred oligo which is not in the original set but from the expanded set, which would be labelled a FP.

**Figure 6:**
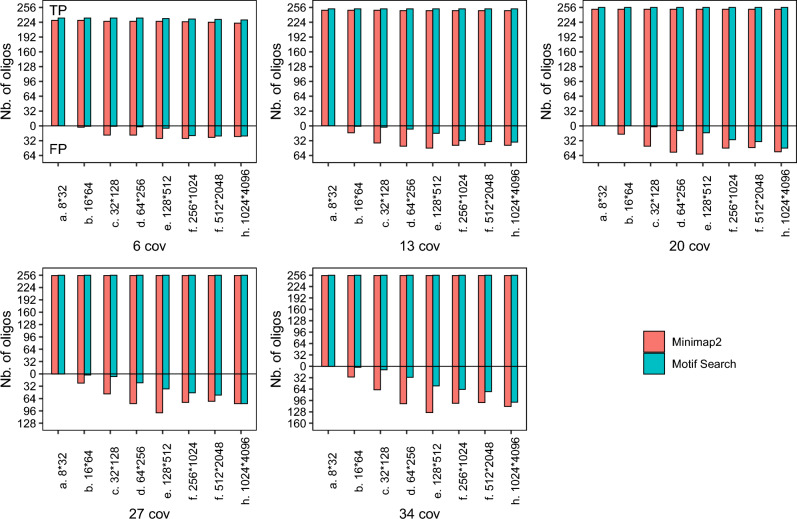
The number of true positive and false positive oligos reconstructed by Motif-Search and Minimap2 for different sequence coverages with expanded motif sets. (i) Motif-Search reconstructs more true positive oligos than reverse alignment even without the knowledge of reference oligos. (ii) False positive rises for both approaches when the motif set size increases.

Figure 6 shows the TP and FP counts for various expanded motif sets. First, note that Motif-Search is able to reconstruct all original oligos when sequence coverage reaches 27× for all motif set sizes. When the sequence coverage is low, Motif-Search is able to reconstruct more true positive oligos than reverse alignment even though it is unaware of the reference oligos. Second, as the motif set size increases, the number of FP for both approaches rise. Since the sequences are error-prone, both approaches make errors identifying the correct references from reads. However, the FP rate of Motif-Search is still lower than reverse alignment. While missing TP is an issue as it can lead to data loss, extra FP is not a problem as it can easily be discarded by using auxiliary metadata and/or error-control coding.

These results clearly demonstrate that (i) our motif-based, BOA method can successfully encode information in DNA, and (ii) with sufficient coverage, Motif-Search is capable of reconstructing all original oligos, and thereby ensuring successful decoding, despite errors introduced by enzymatic BOA and DOS.

### Read–write cost comparison

The cost of storing data on DNA comes from two aspects, namely, the cost of sequencing for reading data and the cost of synthesis for writing data. Composite motifs has the potential to reduce the synthesis cost, thanks to the increase in logical density. For example, each synthesis cycle encodes 84 bits ($log_2{C(96, 32)}$) in our composite motif experiment. A native motif-by-motif approach can solely achieve an encoding of 6 bits per cycle using the same 96 motifs. The conventional phosphoramidite approach has the potential to scale up encoding from 2 bits to as high as 6.38 bits per cycle, as illustrated in the simulation where composite letters are employed for encoding^12^. This 14–42× increase in logical density will lead to a proportionate reduction in synthesis cost over conventional synthesis approaches, as fewer synthesis cycles and fewer oligos are required to encode the same digital data. Since current motif-based synthesis techniques already use a high degree of sequence multiplicity, composite motifs can be easily integrated by generating a variant motif mixture pool without much added costs. The physical density of our approach is 3.36 bits/nt, which is also higher than the physical density of conventional base-by-base DNA storage solutions (2 bits/nt) and sligntly worse than the composite letters approachs (3.37–5 bits/nt^12,13^).

**Figure 7:**
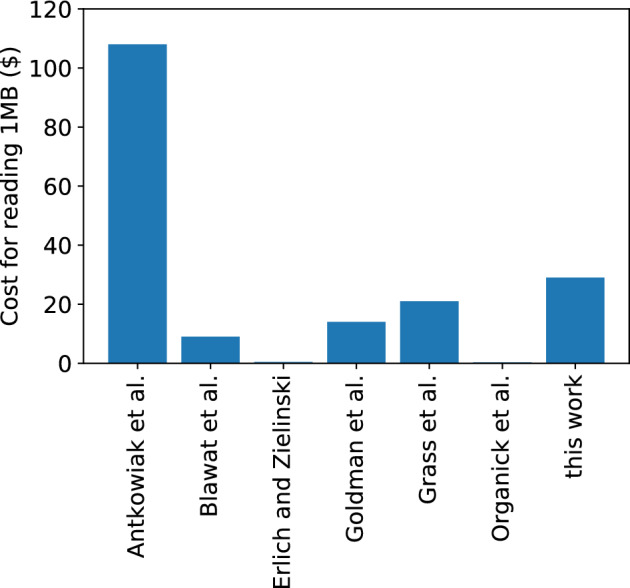
The cost of DNA sequencing to read 1 megabyte data. Our work increases read cost compared to prior work except Antowiak et al.^21^.

Composite motifs offer a distinct advantage in terms of sequencing cost when compared to the composite letters approach^12,13^. Composite motifs necessitate a sequencing coverage of only 20× for complete data recovery, in contrast to the 100–250$$\times$$ required by the composite letters approach. However, in comparison to other non-composite DNA storage systems which require 5× to 10× coverage^14^, our approach trades off read costs for substantial reduction in synthesis costs by increasing logical density. Figure 7 presents a comparison of the cost to read 1MB of data stored in DNA of our approach and other related work^5,7,10,19,21,24^ based on the cost of DNA sequencing (0.006$ per megabase) reported by National Human Genome Research Institute (NHGRI) in August 2021^25^. The detailed calculation is included in Supplementary Table 4. Clearly, our work increases read cost compared to prior work except Antowiak et al. This is expected, as these prior approaches to DNA storage are able to fully recover the data at much lower sequencing coverages of 5× and 10× due to (i) the use of low-error rate array synthesis and high-throughput sequencing with extensive library preparation, and (ii) the use of error-control coding. Our current work, in contrast, focuses on (i) tolerance to errors introduced by enzymatic ligation and direct sequencing without any library preparation , and (ii) complete recovery without additional error correction. As synthesis is approximately 80,000 times more expensive than sequencing^7^ and the read cost continues to drop due to rapid advances in sequencing, we believe that it is more important to focus on reducing the write cost, which is a bottleneck today in DNA data storage.

## Discussion

Today, oligonucleotide synthesis is the dominating bottleneck in making large-scale DNA storage feasible. With the current phosphoramidite synthesis approaches, DNA storage costs several thousands of dollars per MB of data stored. In this work, we demonstrated the feasibility of using composite motifs to scale the logical density of DNA storage by an order of magnitude. We developed synthesis (BOA) and sequencing (DOS) methods customized for writing and reading oligos that regard composite motifs as building blocks, and showed that the error characteristics of these methods are different compared to state-of-the-art techniques. We developed a new motif-based consensus calling and oligo inference method (Motif-Search) that is able to recover all data at coverage as low as 20$\times$. Our future work aims to scale up the methods presented in this paper on several fronts. First, to simplify the task of motif design, we built on an existing library of 25nt primers leading to a physical density of 3.36 bits/nt. Future work will improve this further by optimizing the motif library. Second, we are working on reducing sequencing costs by adding error-control coding optimized to our DNA storage channel to enable data recovery at a lower sequencing coverage. Third, the short size of motif library, the library-preparation-free sequencing provided by DOS, and the error-tolerant nature of Motif-Search all simplify end-to-end automation. Thus, we are developing a fully automated DNA storage solution that can scale both oligo length and number of oligos beyond what was presented in this work to be able to achieve preliminary read and write throughput of Kilobits/second in the near future, and Megabits/second with large-scale parallelization later. With these techniques, we expect our motif-based, enzymatically-ligated DNA storage to become economically feasible for large scale, deep data archival in the near future.

## Methods

### DNA assembly

Oligo with a format of $A_0$–$P_0$ (Fig. 3b) was realised with (i) a set of 8 ssDNA oligo sequences of 24-bases in length, representing $A_0$; and (ii) a set of 32 ssDNA oligo sequences of 50-bases in length, representing the common spacer motif and each $P_0$ motif. The sequences of motifs in these oligos were selected from 25mer DNA barcodes. A set of 8 ssDNA oligo sequences of 50-bases in length were designed to function as (i) a bridge between $A_0$ and $P_0$ for ligation; and (ii) an adenosine overhang on the 3′ end to facilitate AMX sequencing adaptor ligation.

#### Phosphorylation

A pool of 32 oligos, representing the common spacer motif and each $P_0$ motif, were 5′ phosphorylated using T4 PNK at a pool concentration of 300 pmol and reaction scale of 50 ul, as per the vendor guidelines at 37 °C for 40 min. A denaturation step was performed to stop the phosphorylation at 65 °C for 20 min.

#### Annealing and ligation

The 8 $A_0$ oligos and 8 bridge oligos are pooled at equimolar concentrations and diluted to 25 uM final pool concentration. DNA assembly mix was prepared out by taking (1) 2 ul of the $P_0$ phosphorylation mix and (2) 0.5 ul of the $A_0$ and bridge pool (12 pmol). The above reaction is incubated at 95 °C for 3 min and gradually cooled to room temperature. 2.5 ul of the assembly mix along with 5 ul AMX from ONT’s LSK-109 kit and 5 ul Blunt/TA mastermix from NEB and incubated for 10 min at room temperature.

### Nanopore seqeuncing

The ligated sample was then loaded into a R9.4.1 MinIon flowcell prepared with FLow Cell Priming Kit EXP-FLP001 and sequenced for 4 h. Basecalling was performed with Guppy (v4.0.15) and Bonito.

### Motif-search algorithm

Motif-Search works in two stages, *inference* and *consensus calling*. In the inference stage, it maps each read to an inferred oligo. During consensus calling, it uses all inferred oligos to produce a consensus set of inferred reference oligos.

#### Inference

The first task performed by Motif-Search is to extract one or more oligos from each read. Recall that an oligo is a set of motifs concatenated by spacers. Motif-Search infers oligos by first locating the spacer positions and then mapping the portions of the read between two spacers to the reference motifs to determine the payload and address motifs. Inference works in three steps: (i) segmentation to locate spacer positions, (ii) mapping to identify reference motifs between spacers, (iii) overlap check to extract only oligos that do not overlap with each other.

##### Segmentation

Segmentation determines the spacer positions. Since all spacers are identical, their candidate positions can be located by k-mer seeding. We convert A, T, C and G into a two-bit equivalent representation and build the index of the spacer by extracting all k-mers of length four (found to be optimal experimentally). To process each read, we extract all 4-mers in the read, lookup the index, and collect positions with an index hit. The positions are adjusted by the offset of the k-mer to get normalized positions.

To eliminate candidate positions with low confidence, we filter out the positions having less than $spacer\_{length}/k$ k-mer votes. As reads are error prone, indels can cause candidate positions that should be identical to differ slightly by a few nucleotides. This could result in candidates receiving fewer votes and failing the filter. Hence, we merge neighboring positions and represent them by a centroid with a combined count. At the end of this stage, we have all candidate positions for all spacers in a read.

In our experiment, each oligo has only one spacer. But in the general case, each oligo can contain multiple spacers. From the structure of the oligo, we know that each oligo with *M* motifs has $M - 1$ spacers, with each spacer being spaced apart by a distance *d* equal to the sum of the motif length and spacer length. In order to accommodate synthesis and sequencing errors, these inter-spacer gaps can be slightly more or less than the motif length depending on indel errors. Thus, we identify all possible chains of $M - 1$ positions which are within an expected distance threshold from each other.

As mentioned earlier, the candidate positions in these chains are approximate, as indel errors can result in observed starting position differing from actual starting position by a few nucleotides. We rectify and refine these positions to tolerate indel errors by using randomized embedding—a technique which has been demonstrated to be a scalable approach for mapping reads to references in genomic sequence alignment^26^. More specifically, for each candidate position, we extract a spacer-length portion of the read at that position and at several positions around that position. We embed each extracted read fragment using a randomized algorithm and compare with the embedded version of the original spacer motif using hamming distance. We select the shifted position with least embed distance as the final candidate position. As the number of candidate positions can be large, the use of embedding helps us to avoid expensive edit distance computations between the read and spacer motif, and use hamming distance between their embedded versions to rectify candidate positions.

##### Mapping

Given a chain of refined candidate positions, we can extract the potion of each read between two neighboring spacers. These portions correspond to address and payload motifs. The next step is to identify the original motif for each observed motif in the read. This can be translated to a sequence mapping problem by considering the original motif library as the reference and the observed motif in the read as the query. Therefore, we use the ksw-lib^27^ to select the optimal original motif with the highest mapping score for each observed motif. After this step, we have multiple chains of mapped motifs.

##### Overlap check

As we consider all possible chains, some chains might overlap each other. However, while each read can cover multiple oligos due to DOS, each nucleotide in a read should map to only one motif/oligo. Thus, the final step in the inference stage is to identify the optimal set of chains that do not overlap with each other. To do this, we traverse the chains to identify overlapping sets. For each overlapping set, we pick a chain with the highest mapping score such that no chain appears in two sets.

#### Consensus calling

**Figure 8:**
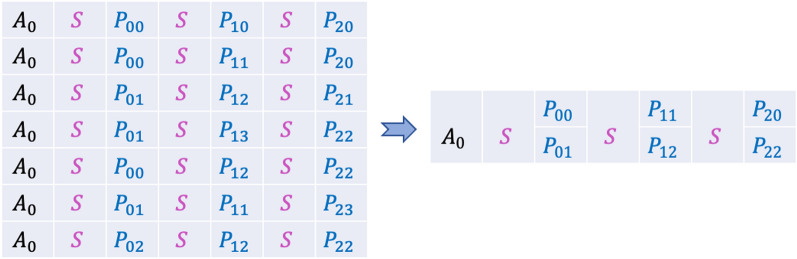
Example showing consensus calling with seven inferred oligos with the same address motif $A_0$ . The payload motifs are decoded as $P_{00}$, $P_{01}$ at the first position, $P_{11}$, $P_{12}$ at the second position and $P_{20}$, $P_{22}$ at the third position which are the *topN* (N is the number of oligos in each sequence) frequent motifs in each column position.

Each original encoded oligo can be synthesized with duplication. Library preparation steps, like PCR, also amplify the pool of oligos by creating multiple copies of each oligo to ensure successful sequencing. Thus, an original oligo can be covered by multiple reads. For each read, the inference stage identifies the optimal set of non-overlapping chains. As the final step, we apply consensus calling to group similar motif chains inferred from the *inference* stage, and obtain consensus to achieve higher confidence. We do this by first clustering the inferred oligos using their address motifs. Then, we select the most frequent motifs at each position as the final consensus motif as shown in Fig. 8.

### Supplementary Information

Supplementary Information.

## Data Availability

The oligo sequences, the reads with Guppy basecalled, Bonito basecalled and Bonito basecalled post-processed by SaberSplit are available via https://drive.google.com/drive/folders/1VPQUAye0BUVUSnePoc8M1mN7GZw09u_1?usp=sharing.

## Code availibility

The Motif-Search algorithm implementation is available via https://gitlab.eurecom.fr/yan1/motif-search under MIT license. SaberSplit is available via https://github.com/helixworks-technologies/sabersplit.
