# Supplementary material for: Scaling logical density of DNA storage with enzymatically-ligated composite motifs
Source: Sci Rep. 2023 Sep 25;13:15978. doi: 10.1038/s41598-023-43172-0 (PMC10519978; doi:10.1038/s41598-023-43172-0)
Supplement: Supplementary file 1 — Supplementary Information. [file 41598_2023_43172_MOESM1_ESM.pdf]

SUPPLEMENTARY INFORMATION

Supplementary Table 1. The substitution (SUB), insertion (INS) and deletion (DEL) rate of SOTA work. Data for Goldman et al. [1], Grass et al. [2], Erlich Zielinski [3], Organick et al. [4] and Antkowiak et al. [5] was taken from Antkowiak et al. [5].

	Goldman et al.	Grass et al.	Erlich et Zielinski	Organick et al.	Antkowiak et al.	This Work
SUB	0.00088	0.005850	0.003870	0.005400	0.026000	0.011411
INS	0.00036	0.000230	0.000211	0.004500	0.057000	0.007817
DEL	0.00036	0.000230	0.000211	0.001500	0.062000	0.007485

Supplementary Table 2. Statistics of the reads.

	Nb. reads	Nb. aligned reads	Median read length
Guppy	27198	9960	166
Bonito	27198	4901	110
SaberSplit	102221	4434	25

Supplementary Table 3. Processing time (in second) for Minimap2-based reverse alignment and Motif-Search with 12 CPUs.

	6×	13×	20×	27×	34×
Motif-Search exec. time	0.15	0.24	0.38	0.49	0.64
Reverse alignment exec. time	29	60	91	122	152

Supplementary Table 4. Sequencing cost projection. The sequencing cost to read 1 Megabyte is simulated as the Equation 1.

$$reading\_cost = oligo\_length * nb\_reads * sequencing\_cost\_per\_nt / stored\_data\_size \quad (1)$$

The oligo_length includes the length of primers. The sequencing_cost_per_nt takes the value 0.006$ per megabase reported by National Human Genome Research Institute (NHGRI) in August 2021. Data for Goldmann et al. [1], Grass et al. [2], Erlich et al. [3] and Organick et al. [4] was taken from Organick et al. [4].

	oligo length	nb of reads	data size	sequence cost ($/MB)
Antkowiak et al.	60	30000000	99103 bit	108
this work	74	640	80 bit	29.8
Grass et al.	159	1858027	679000 bit	21.9
Goldman et al.	183	7960000	5200000 bit	14.1
Blawat et al.	230	144475005	22 MB	9.06
Erlich and Zielinski	200	750000	2.11 MB	0.43
Organick et al.	150	67241860	200 MB	0.3

Supplementary Note 1. Sequencing data and SaberSplit.

The generated sequences via the BOA method were sequenced via DOS for 4 hours to generate 27,198 reads with an N50 of 192bp. The reads were basecalled using guppy basecaller (v4.0.14) in high accuracy mode. We suspected that some of the reads would not be split properly by the MinION instrument as observed in prior research. To split the concatenated reads and to improve basecalling, we developed a nodejs script called SaberSplit to correctly identify the adapter regions of the reads, which has an unique pA (picoamperage) signature that can be identified from the events data of the fast5 file.

SaberSplit is a node.js based tool to process the .TSV files generated from the SquigglePull program of the SquiggleKit(https://github.com/Psy-Fer/SquiggleKit). SquigglePull TSV files contain the read-IDs and their event level data in the following format. SaberSplit extracts the event data from the TSV files and stores them in an array. It calculates the median and MAD (Median Absolute Deviation) of the event data. It calculates (Datai-Median)/MAD for each of the data points and if the (Datai-Median)/MAD > 5. It takes that data point for further processing.

SaberSplit extracts the events on the right-hand and left-hand side of the triggered event if they have (Datai-Median)/MAD > 3. If the total number of extracted events that are on the left and right along with the triggered event is less than 12 events. The event is classified as a spike and the read is split. SaberSplit processed 27,198 reads, generating 237,327 new split reads. Many of the split reads are short and have not passed the threshold for basecalling with bonito. A total of 102,222 were successfully basecalled from the SaberSplit reads. Guppy generated an extremely low number of successful basecalled reads from the SaberSplit reads.

Supplementary Note 2. Minimap2 parameterization.

We opt for the “ONT” mode and set the kmer value to 6 for performing the reverse alignment. Notably, the default kmer in Minimap2 is 15. We tried various kmer sizes including 14, 12, 10, 8, and 6 within our tests. Our primary focus is on the capability of Minimap2 to identify all the original oligos even with the knowledge of the original oligos. That’s why we prompted our choice of kmer=6 to maximize the retrieval of oligos despite the potential for a slower process. However, even with kmer=6, Minimap2 does not succeed in locating all the original oligos, and its trade-off between true positives (TP) and false positives (FP) is less favorable compared to Motif-Search. This discrepancy highlights the comparative performance of Motif-Search in our analysis.

References

[1] Nick Goldman, Paul Bertone, Siyuan Chen, Christophe Dessimoz, Emily M LeProust, Botond Sipos, and Ewan Birney. Towards practical, high-capacity, low-maintenance information storage in synthesized dna. *nature*, 494(7435):77–80, 2013.

[2] Robert N Grass, Reinhard Heckel, Michela Puddu, Daniela Paunescu, and Wendelin J Stark. Robust chemical preservation of digital information on dna in silica with error-correcting codes. *Angewandte Chemie International Edition*, 54(8):2552–2555, 2015.

[3] Yaniv Erlich and Dina Zielinski. Dna fountain enables a robust and efficient storage architecture. *science*, 355(6328):950–954, 2017.

[4] Lee Organick, Siena Dumas Ang, Yuan-Jyue Chen, Randolph Lopez, Sergey Yekhanin, Konstantin Makarychev, Miklos Z Racz, Govinda Kamath, Parikshit Gopalan, Bichlien Nguyen, et al. Random access in large-scale dna data storage. *Nature biotechnology*, 36(3):242–248, 2018.

[5] Philipp L Antkowiak, Jory Lietard, Mohammad Zalbagi Darestani, Mark M Somoza, Wendelin J Stark, Reinhard Heckel, and Robert N Grass. Low cost dna data storage using photolithographic synthesis and advanced information reconstruction and error correction. *Nature communications*, 11(1):1–10, 2020.
